# Supplementary material for: Ultrasensitive mechanical detection of magnetic moment using a commercial disk drive write head
Source: Nat Commun. 2016 Sep 20;7:12714. doi: 10.1038/ncomms12714 (PMC5034305; doi:10.1038/ncomms12714)
Supplement: Supplementary Information — Supplementary Figure 1-9 and Supplementary Note 1-3 [file ncomms12714-s1.pdf]

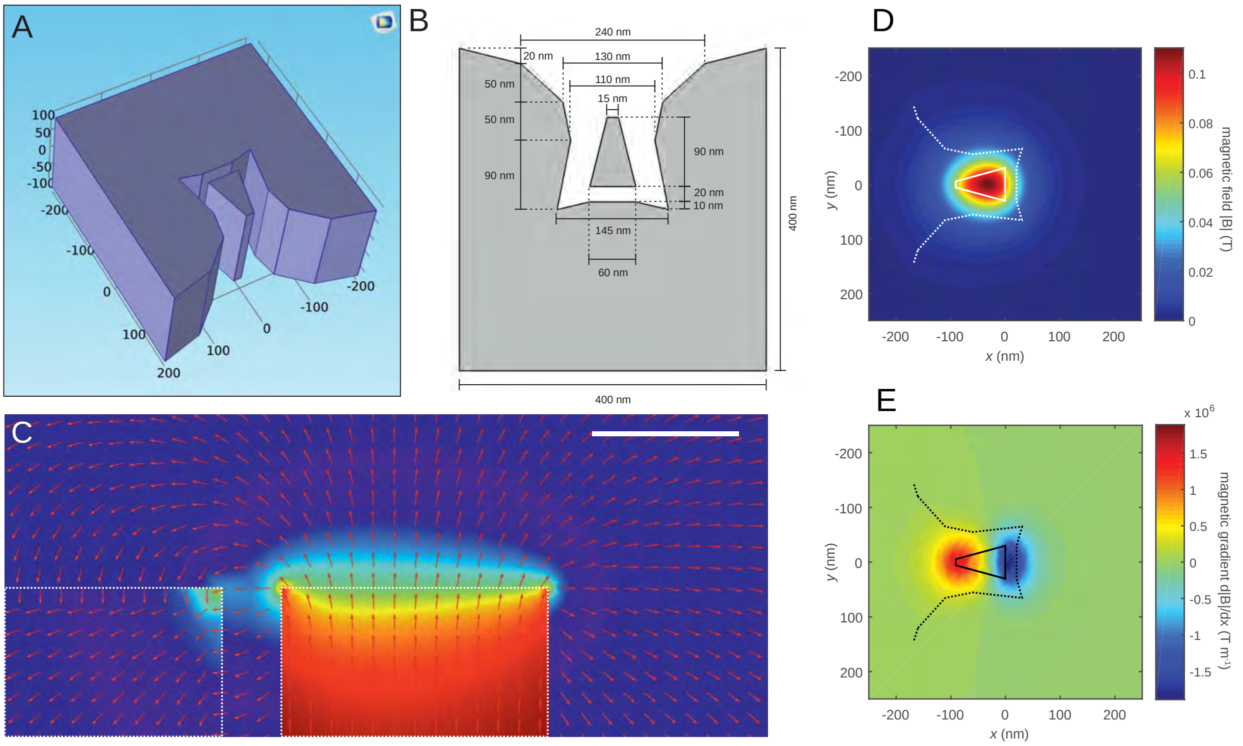

Supplementary Figure 1: **Finite element simulations performed with COMSOL.** (A) The model consists of a write pole and a return shield surrounded by air. (B) The in-plane geometry was extracted from a scanning electron microscope image (Fig. 1d of the main text) while the thickness of the return shield was estimated from a cross-sectional image obtained using focused ion beam machining. (C) Profile cut showing the simulated magnetic field at $y = 0$. The absolute value of B is in indicated in color, ranging from 1 T (red) regions to 0 T (deep blue). Arrows with normalized length show the direction of the field at the position of their tail. Geometrical boundaries are traced as white dotted lines. Scale bar is 50 nm. (D) Magnetic field and (E) magnetic gradient as xy plot at a distance of 30 nm from the writer surface and for a pole magnetization of 0.87 T (corresponding to 5 mA drive current). Solid contour is the write pole and dashed contour is the trailing shield.

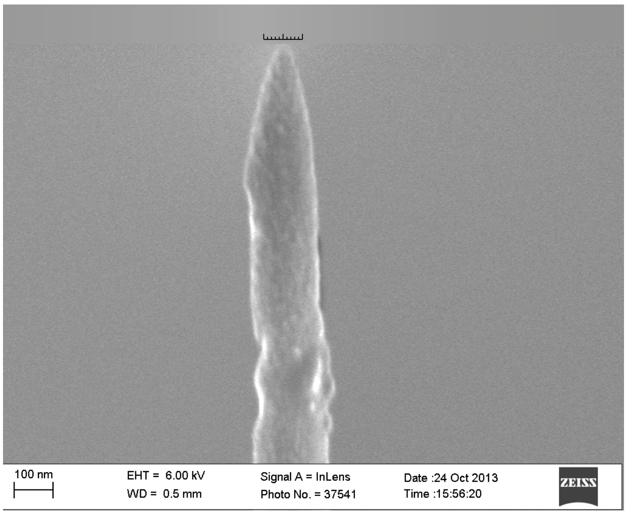

Supplementary Figure 2: **Dimensions of tip A.** Scanning electron micrograph of Pt/YF_3-coated nanowire. Ruler is 10 nm per tick. The estimated tip width is 40 nm.

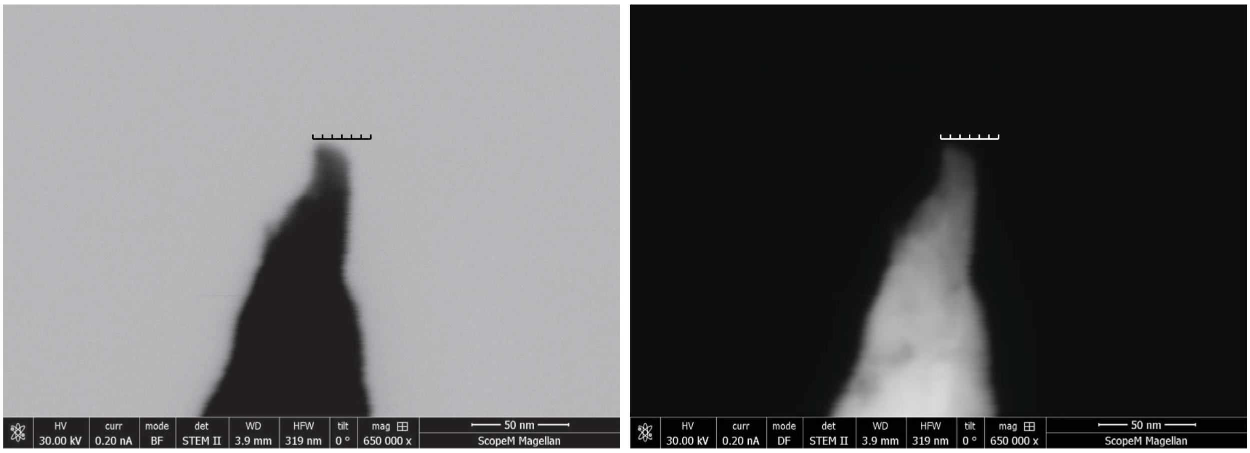

Supplementary Figure 3: **Dimensions of tip B.** Bight and dark field scanning electron micrographs of bare diamond nanowire tip. Ruler is 5 nm per tick. The estimated tip width is 18 nm.

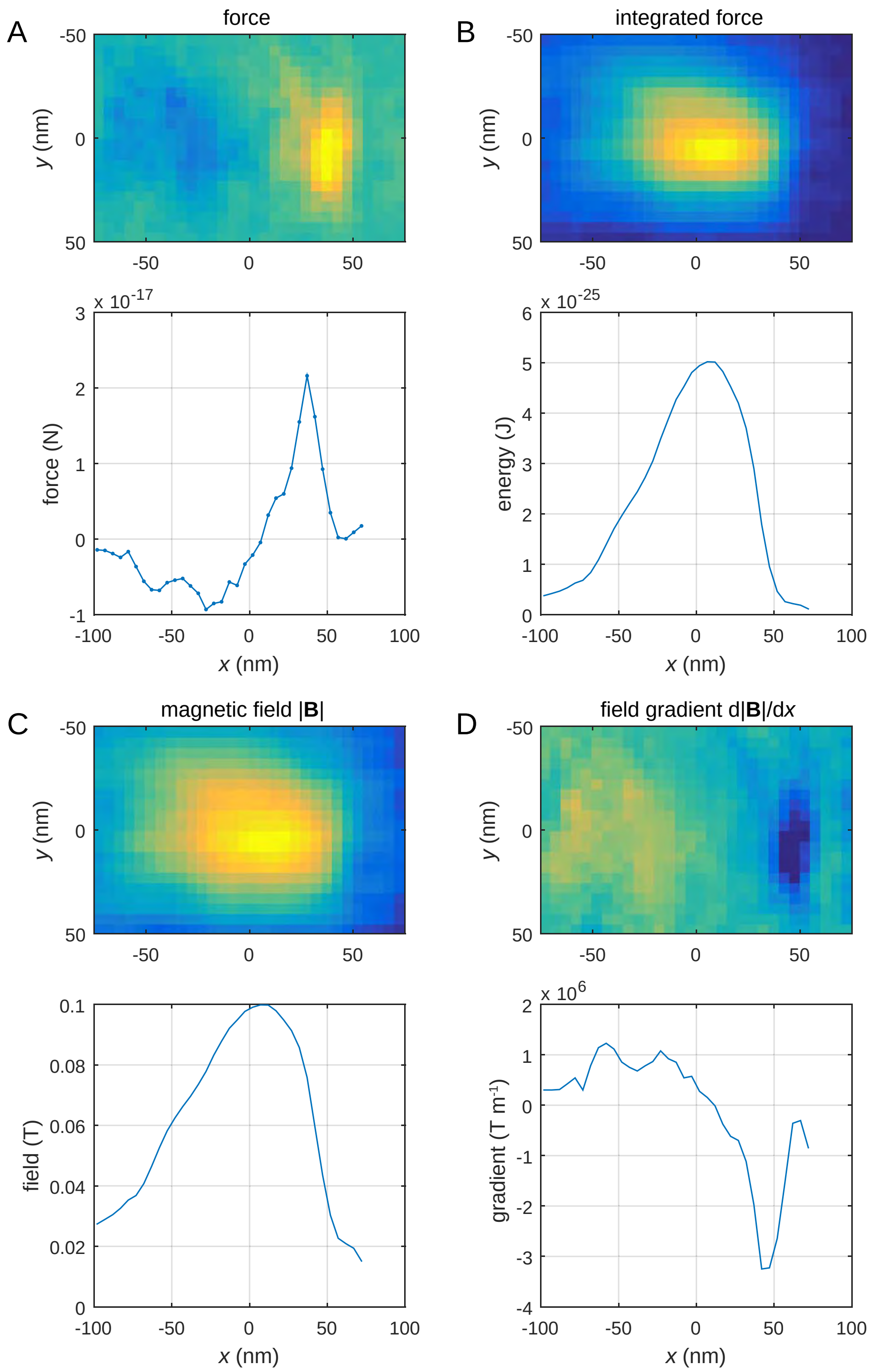

Supplementary Figure 4: **Step-by-step field analysis.** Two-dimensional and one-dimensional maps of force (A), magnetostatic energy (B), magnetic field (C) and field gradient (D). The one-dimensional maps are obtained by integrating the corresponding two-dimensional plots from $y = -18\,\mathrm{nm}$ to $y = 17\,\mathrm{nm}$.

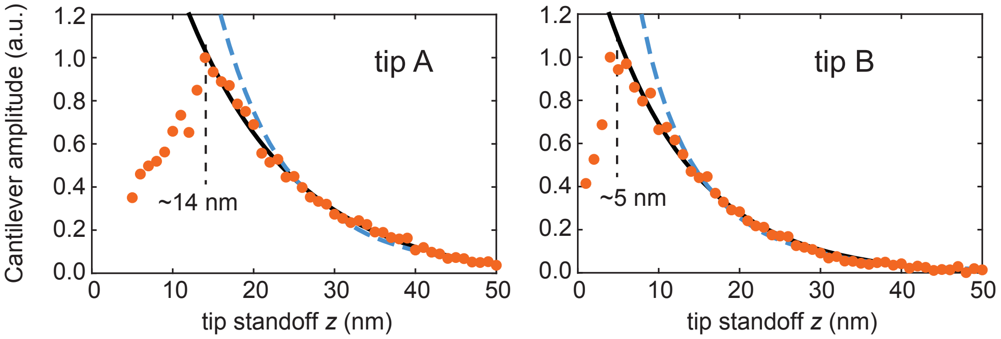

Supplementary Figure 5: **Approach curves for both tips.** The solid lines are exponential fits to the points with $z > 14\,\mathrm{nm}$ (tip A) and $z > 5\,\mathrm{nm}$ (tip B). The dashed lines are from the finite element modeling. The tip B data are shown in Figure 5c in the main manuscript. Both curves were recorded over the xy location with maximum signal and with a $12\,\mathrm{mA}$ drive current.

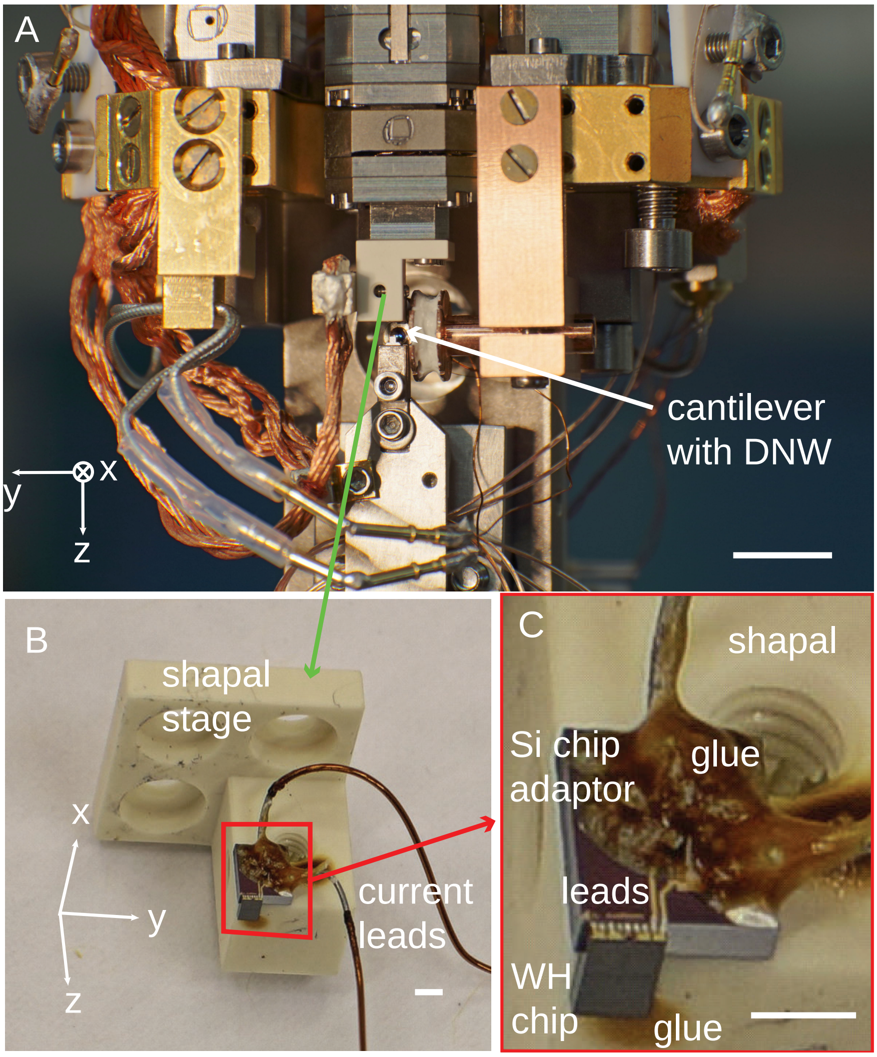

Supplementary Figure 6: **Integration of an isolated write head into a scanning microscope system.** (A) The MRFM probe used in this study, shown here fully equipped with cantilever sensor and WH on shapal sample stage. (B) and (C) zoom-in photographs of a mounted WH (See text for details of assembly). Scale bars are 10 mm, 1 mm, and 1 mm in (A)-(C), respectively.

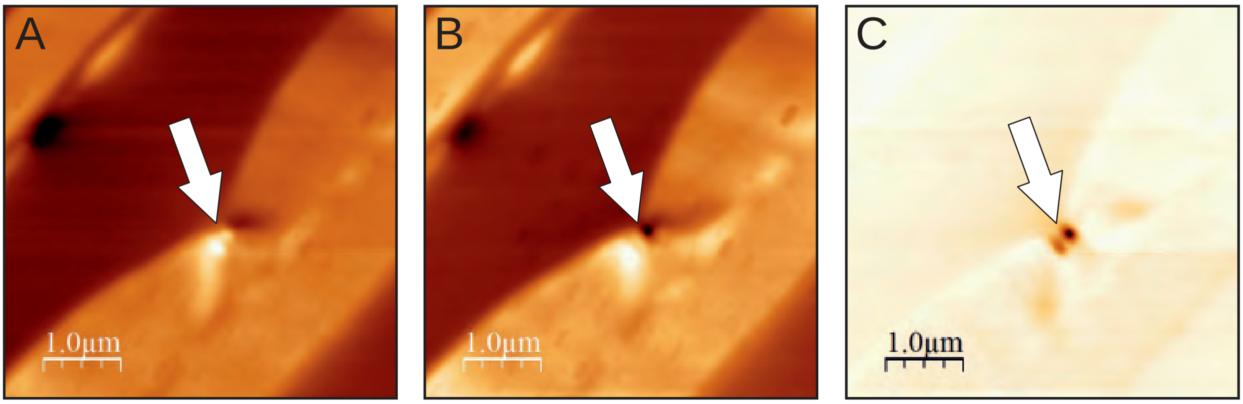

Supplementary Figure 7: Magnetic force microscopy images of finished device. We used an Asylum Cypher MFM to confirm electrical control of the write pole magnetization. The signal is qualitatively proportional to the sample stray field gradient (normal to the surface) that interacts with the magnetized probe tip. The probe was scanning 50 nm above the surface to minimize topographical effects. (A) Scan with a static current of 5 mA applied to the write head coil. (B) Scan with −5 mA, showing inverted contrast at the position of the write pole. The two scans show that the magnetization of the sample is independent of the applied voltage except for the magnetization of the write pole itself, whose sign inverts along with the applied voltage (arrow). (C) Difference between the images in (A) and (B).

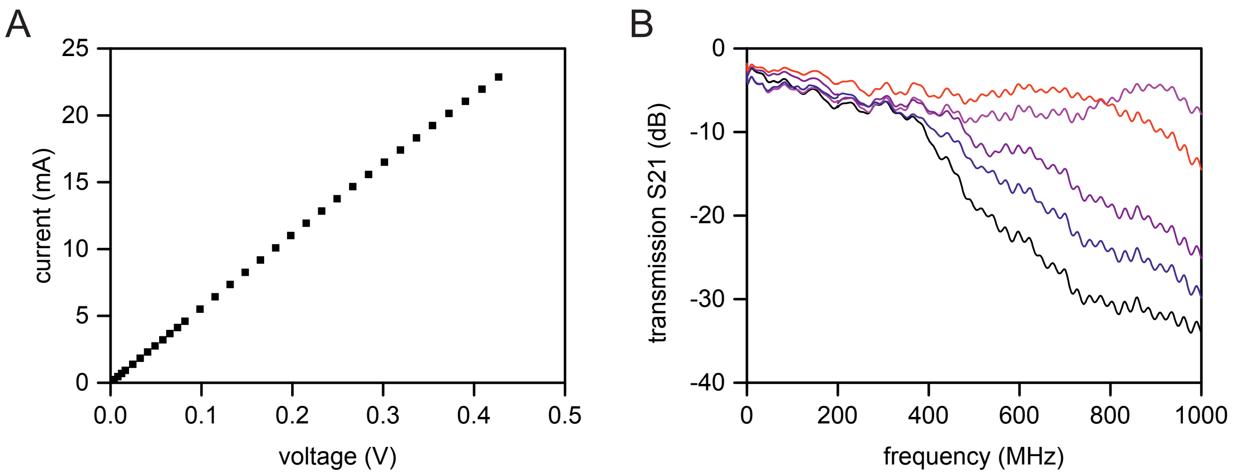

Supplementary Figure 8: **Electrical characteristics of write head coils.** Before using write heads in a scanning probe experiment, we characterized their electrical properties. We found that reconnected WH devices showed linear voltage-current relationship and typically had an electrical resistance of $20 - 40\,\Omega$ at room temperature and of about $10 - 20\,\Omega$ at $4\,$K. The resistance of the write head drive coil is specified around $3\,\Omega$ at room temperature (likely lower at cryogenic temperatures); the main contribution to the resistance comes from the epoxy contacts and the leads on the jumper chip. (A) Voltage-current trace of a write head device submerged in liquid helium. The resistance is about $18\,\Omega$ for this particular device. (B) Forward transmission (S21) of several write head devices at room temperature. We believe that transmissions below $-10\,$dB are mostly caused by the limited quality and consistency in electrical contact that is achievable using conductive epoxy. We expect that the radio frequency characteristics of reconnected write heads can be improved by using wire bonds and an impedance-matched jumper piece.

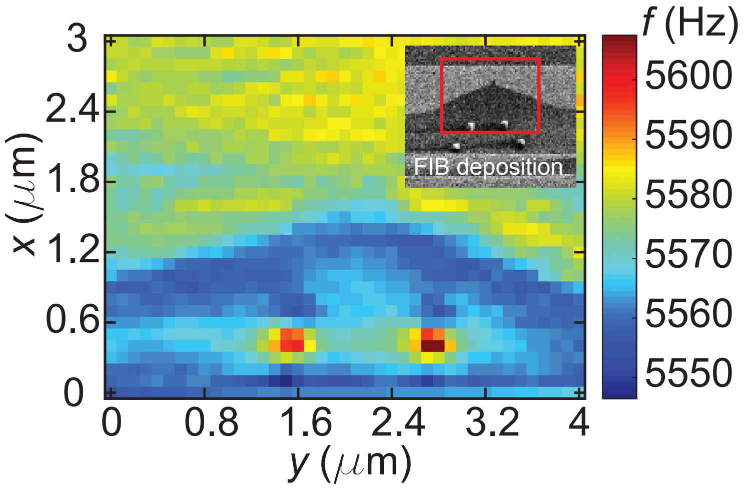

Supplementary Figure 9: **Sample Positioning.** The inset shows an FIB image of a write pole area with deposited Pt marker dots. The main figure shows a frequency scan of the area in the red rectangle using a diamond nanowire-tipped silicon cantilever.

Supplementary Note 1: Estimation of tip volume

The volume of the bare diamond nanowire tip (tip B) was estimated from two high-resolution scanning electron micrographs (Supplementary Figure 3). From these images we estimated a tip diameter of $D = 18\,\text{nm}$. For simplicity, we assume in our model that the tip is cylindrical with an axis parallel to the z direction.

To calculate the total force (amplitude) acting on the tip, we integrated the position-dependent force over the entire volume of the cylinder. The force exerted on a volume element dV at location $\mathbf{r}$ is

$$dF(\mathbf{r}) = F_0(\mathbf{r})dV \ , \tag{1}$$

with

$$F_0 = \frac{\chi}{\mu_0}\nabla|\mathbf{B}(\mathbf{r})|^2 \ . \tag{2}$$

To obtain the total (integrated) force we assume that the force is uniform over the cylinder cross-section $\pi(D/2)^2$ and thus only depends on z. This is a good approximation, because the diameter of the nanowire is very small. Moreover, from Figure 5 c in the main manuscript we know that the force decays exponentially with distance with decay length δ. The total force is

$$\begin{aligned}
F &= \int_{\text{cylinder}} dV F_0(\mathbf{r}) && (3)\\
&= \pi(D/2)^2 \int_z^\infty dz' F_0(z') && (4)\\
&= \pi(D/2)^2 \int_z^\infty dz' F_0(0) e^{-z'/\delta} && (5)\\
&= \pi(D/2)^2 \delta F_0(z) && (6)\\
&= \frac{\chi\pi(D/2)^2\delta}{\mu_0}\nabla|\mathbf{B}(z)|^2 && (7)
\end{aligned}$$

where z is the tip standoff. Comparing with Eq. (1) in the main manuscript,

$$\mathbf{F}(\mathbf{r}) = \nabla[\boldsymbol{\mu}(\mathbf{r})\cdot\mathbf{B}(\mathbf{r})] = \frac{\chi V}{\mu_0}\nabla|\mathbf{B}(\mathbf{r})|^2 \ , \tag{8}$$

we notice that the force on the cylinder is equivalent to the force on a point-like particle located at distance z and with effective tip volume $V_{\text{eff}} = \pi(D/2)^2\delta$. For tip B, $D = 18\,\text{nm}$ and $\delta = 11.3\,\text{nm}$, resulting in $V_{\text{eff}} = 2.88 \times 10^{-24}\,\text{nm}^3$.

Since the tip diameter is very small, it is difficult to read off a precise tip size from the micrographs in Supplementary Figure 3, and moreover, the actual tip geometry is not

exactly cylindrical. We have therefore estimated upper and lower bounds of the tip volume. As an upper bound, we assume a cylindrical tip with a diameter of 20 nm, resulting in $V_{\text{eff}} = 3.55 \times 10^{-24}\,\text{nm}^3$. As a lower bound, we assume a tip with a diameter of 16 nm and an apex radius of 8 nm, resulting in $V_{\text{eff}} \approx 1.51 \times 10^{-24}\,\text{nm}^3$. This corresponds to an error of $+23/-48\%$. The error in the magnetic field and field gradient is smaller, because they scale as $\propto V^{-1/2}$. The resulting upper and lower bounds for $|\mathbf{B}|$ and $\partial_x|\mathbf{B}|$ are $+28/-11\%$.

Due to the small size of the tip and its large surface-to-volume ratio, it is important to consider possible contributions to the measured force from surface adsorbates including water and hydrocarbon molecules, which are known to form a 1 nm-thick coating on most solid surfaces [1]. The susceptibilities of water, ice, and hydrocarbons are $9 \cdot 10^{-6}$, $6.4 \cdot 10^{-7}$, and $8.2 \cdot 10^{-7}$ [2, 3], respectively, and are thus much lower than that of diamond, $-2.2 \cdot 10^{-5}$. In addition, the total volume of these adsorbate materials are substantially smaller than the diamond tip volume. We therefore conclude that common surface adsorbates contribute negligibly to the measured force signal.

Supplementary Note 2: Calculation of magnetic field and field gradient from force values

The images of magnetic field and field gradient in Figure 5 a,b and the values reported in Table 1 were determined from the force map shown in Figure 4 b.

In a first step, a map of the magnetostatic energy W was calculated according to Eq. (3) in the main manuscript. For each y line, the force was numerically integrated from right to left (in negative x direction) resulting in a map for W. We have integrated in $-x$ direction because the field at the $+x$ edge is essentially zero while the field is still slightly present at the $-x$ edge. In a next step, we calculated the magnetic field by scaling with $\mu_0/(\chi V)$ and taking the square root. The volume was estimated from scanning electron micrographs (Supplementary Figures 2 and 3) and the χ value was set to the value of bulk diamond (see Methods). The gradient was calculated by taking a numerical derivative in x-direction.

We have also evaluated the magnetic field and field gradient as a one dimensional line scan. For this purpose we have averaged the force map in y over the central region (from $y = -18... + 17\,\text{nm}$) and then performed the procedure described above. From the one-dimensional scans we could read off the peak field (92 mT) and peak gradient ($3.0\,\text{MTm}^{-1}$).

These values were measured at a tip standoff of 30 nm and a drive current of 5 mA.

The numerical values reported in Table 1 are the above peak field and peak gradient values, extrapolated to a tip standoff of 5 nm and drive currents of 12 mA and 30 mA. For the extrapolation, we used the scaling implied by Figures 5 c,d. For the tip standoff z, this scaling is $F(z) = \exp[-(z - 5\,\mathrm{nm})/11.3\,\mathrm{nm}]$. (To a very good approximation, the same exponential scaling also applies to $|\mathbf{B}|$ and $\partial_x|\mathbf{B}|$ as confirmed by the finite element model). For the drive current, the scaling is given by Supplementary Equation (10). Since all of the above measurements were performed with the same tip and the same write head device, this extrapolation is expected to be accurate.

Supplementary Note 3: Calibration of the write pole magnetization

We have used the force versus drive current plot (Figure 5 d in the main manuscript) to calibrate the pole magnetization. In a first step, we have assumed that the pole magnetization follows a Langevin function with drive current I,

$$M_{\mathrm{pole}}(I) = M_{\mathrm{sat}} \left[\coth\left(\frac{I}{I_c}\right) - \frac{I_c}{I}\right] \tag{9}$$

M_{sat} is the saturation magnetization which we assume to be equal to the saturation magnetization of FeCo ($\mu_0 M_{\mathrm{sat}} = 2.4\,\mathrm{T}$, [4]). The only unknown parameter in Supplementary Equation (9) is the knee current I_c.

We next determined I_c by fitting the force curve in Figure 5 d. For this purpose, recall that the amplitude of the measured force F_0 in response to a sinusoidal driving current $I(t) = I_0 \sin(\pi f_c t)$ of amplitude I_0 is given by

$$F_0(I_0) = \frac{\chi V}{\mu_0 T_c} \int_0^{T_c} dt \cos(2\pi t f_c) B[I(t)]^2 \propto \int_0^{T_c} dt \cos(2\pi t f_c) M_{\mathrm{pole}}[I(t)]^2 := F_a(I_0) + F_b \tag{10}$$

We have used a non-linear fitting algorithm (Matlab, nlinfit) to match the above equation to the plot in Figure 5 d. The fit had three free parameters: The overall amplitude of the force F_a, an additional offset F_b to account for a baseline, and the knee current I_c. The resulting fit parameters were $F_a = 674 \pm 67\,\mathrm{aN}$, $F_b = 32 \pm 9\,\mathrm{aN}$ and $I_c = 10.7 \pm 1.0\,\mathrm{mA}$ in 95% confidence. By using Supplementary Equation (9) one finds a pole magnetization $\mu_0 M_{\mathrm{pole}}$ of $370 \pm 35\,\mathrm{mT}$ at $I_0 = 5\,\mathrm{mA}$, a magnetization of $830 \pm 70\,\mathrm{mT}$ at $I_0 = 12\,\mathrm{mA}$, and

a magnetization of $1565 \pm 75\,\mathrm{mT}$ at $I_0 = 30\,\mathrm{mA}$.

---

[1] Degen, C. L., Poggio, M., Mamin, H. J., Rettner, C. T. & Rugar, D. Nanoscale magnetic resonance imaging. *Proc. Nat. Acad. Sci. U.S.A.* **106**, 1313 (2009). URL http://dx.doi.org/10.1073/pnas.0812068106.

[2] Senftle, F. & Thorpe, A. Oxygen adsorption and the magnetic susceptibility of ice at low temperatures. *Nature* **194**, 673 (1962). URL http://dx.doi.org/10.1038/194673a0.

[3] Balta-Calleja, F. J., Berling, K. D., Cackovic, H., Hosemann, R. & Loboda-Cackovic, J. Diamagnetic susceptibility of solid and liquid paraffins. *J. Macromol. Sci. B* **12**, 383–392 (1976). URL http://dx.doi.org/10.1080/00222347608019326.

[4] Bardos, D. I. Mean magnetic moments in bcc fe-co alloys. *J. Appl. Phys.* **40**, 1371–1372 (1969).
